# Lack of Identification in Semiparametric Instrumental Variable Models With Binary Outcomes

**DOI:** 10.1093/aje/kwu107

**Published:** 2014-05-23

**Authors:** Stephen Burgess, Raquel Granell, Tom M. Palmer, Jonathan A. C. Sterne, Vanessa Didelez

**Keywords:** Avon Longitudinal Study of Parents and Children, generalized method of moments, identifiability, identification, instrumental variables, semiparametric methods, structural mean model, weak instruments

## Abstract

A parameter in a statistical model is identified if its value can be uniquely determined from the distribution of the observable data. We consider the context of an instrumental variable analysis with a binary outcome for estimating a causal risk ratio. The semiparametric generalized method of moments and structural mean model frameworks use estimating equations for parameter estimation. In this paper, we demonstrate that lack of identification can occur in either of these frameworks, especially if the instrument is weak. In particular, the estimating equations may have no solution or multiple solutions. We investigate the relationship between the strength of the instrument and the proportion of simulated data sets for which there is a unique solution of the estimating equations. We see that this proportion does not appear to depend greatly on the sample size, particularly for weak instruments (ρ^2^ ≤ 0.01). Poor identification was observed in a considerable proportion of simulated data sets for instruments explaining up to 10% of the variance in the exposure with sample sizes up to 1 million. In an applied example considering the causal effect of body mass index (weight (kg)/height (m)^2^) on the probability of early menarche, estimates and standard errors from an automated optimization routine were misleading.

When fitting a statistical model, parameter estimates are usually obtained by the optimization of an objective function. For example, in linear regression, parameters are chosen such that the sum of the squared differences between the fitted and the measured values of the response variable at each data point is minimized. In this case, the sum of the squared differences is the objective function. Provided that the data are not in some way degenerate (e.g., the value of a covariate in the model is the sum of 2 other covariates, an example of collinearity), there is a set of parameter values that is a unique solution to this optimization, where the objective function achieves its minimal value. In this case, we say that the parameters are identified.

In causal inference, identifiability is obtained if a causal parameter can, in principle (i.e., from infinite data), be uniquely determined on the basis of the distribution of observable variables (1). In this paper, we widen this definition to consider the identification of parameters in finite samples. This is because there is not much practical utility in knowing that an estimating equation asymptotically gives a unique solution if it does not do so for realistically sized samples. Lack of identification occurs when an objective function used for parameter estimation is not optimized at a single parameter value, but rather multiple values of the parameter satisfy the optimization criteria (2). This problem would occur, for example, if sex were included as a covariate in a model for a population that is all female. Because there are no men in the population, the parameter value for men can take an arbitrary value; there is no information on the parameter in the data set. In such a case, most automated optimization routines for fitting statistical models within a software package would give some indication that an error had occurred.

However, lack of identification may be more subtle, particularly outside the world of linear models. Even if a parameter is formally identified and there is a unique optimal parameter estimate, estimation may be problematic. For example, if an objective function is not shaped similar to the letter U, but rather similar to the letter W, then the function will have 2 local minima, 1 of which may not be the global minimum. An optimization routine may find a local minimum rather than the global minimum first depending on the arbitrary choice of starting values for the optimization and thus fail to report the true optimal parameter values.

Alternatively, the identification may be weak. Weak identification occurs when the gradient of the objective function is close to 0 in the vicinity of the optimal estimate (3). This means that multiple values of the parameter almost satisfy the optimization criteria. Even if the parameter estimates are formally identified, the data do not carry much information on the true value of the parameter. Asymptotically derived standard errors and confidence intervals, which typically assume that the objective function is well behaved (formally, that it can be approximated by a quadratic function in the neighborhood of the optimal value) may be underestimated.

Here, we consider the issue of parameter identification in instrumental variable (IV) estimation with a binary outcome. An IV is a variable that can be used to estimate the causal effect of an exposure on an outcome from observational data (4). The claim of causal inference depends on the following 3 assumptions about the IV, exposure, and outcome distributions: 1) the IV is associated with the exposure; 2) the IV is not associated with any confounder of the exposure-outcome association; and 3) the IV is conditionally independent of the outcome given the exposure and confounders of the exposure-outcome association (5, 6).

The third condition implies that the IV cannot directly affect the outcome other than through the exposure (i.e., the exclusion restriction assumption (4)). Although these assumptions allow the null hypothesis of no causal effect of the exposure on the outcome to be tested, further assumptions are needed to estimate the causal effect of the exposure on the outcome (7). The specific assumptions required depend on the method used for estimation (8). In the linear case, problems of weak identification have been observed when the IV explains a small proportion of the variation in the exposure (9). Such IVs have been associated with bias and poor coverage properties (e.g., inflated type I error rates) in finite samples (10, 11). In the binary outcome case, little is known about the potential problems of poor identification.

Instrumental variable methods have risen to prominence in the epidemiologic literature over the past decade under the banner of Mendelian randomization (12). Mendelian randomization is the use of genetic variants as IVs (13). Genetic variants are ideal candidate IVs, because genes typically have specific functions, and the genetic code for each individual is fixed at conception, meaning that variants cannot be influenced by social or environmental factors (14). Although the findings of this paper are not limited in application to Mendelian randomization, the parameter values and examples chosen reflect those typical of Mendelian randomization investigations. In particular, the proportion of the variance in risk factor exposures explained by known genetic variants is often low (15). Different conclusions may therefore be reached in other areas of application, such as the use of IVs for estimating the causal effect of the treatment in a randomized trial (16).

In this paper, we consider multiplicative structural models for binary outcomes. In the econometrics literature, these are typically fitted by the generalized method of moments (GMM) (17), whereas in the medical statistics literature, a slight variation of these models, known as structural mean models (SMMs) (18) is usually fitted by the method of g-estimation (we refer to this as the SMM method for simplicity of presentation). These methods are known as semiparametric, because they do not make full distributional assumptions, but only specify the structural mean relationship between the exposure and outcome (19). We contrast these with the fully parametric 2-stage method, the binary-outcome analog of the 2-stage least squares method commonly used in IV estimation with continuous outcomes (20). Although other methods are available for IV estimation with a binary outcome (21), we focus on these methods in this paper, because they are the most commonly used in applied practice and are available in standard statistical software packages. Although we compare these methods with regard to the issue of identification, this paper is not a comprehensive evaluation of methods for instrumental variable analysis with a binary outcome and should not be used out of context as a pretext for preferring 1 analysis method over another.

The structure of this paper is as follows: first, we introduce the GMM, SMM, and 2-stage methods and exemplify the problem of lack of identification in an applied example; we then perform a simulation study to investigate the frequency of poor identification (no or multiple parameter values satisfying the estimating equations) for different sample sizes and strengths of the IV, measured as the proportion of variance in the exposure explained by the IV; finally, we illustrate the consequences of poor identification with real data analyzed using an automated optimization routine to demonstrate the potential for poor identification to give misleading inference in an applied context.

## METHODS

We assume a log-linear structural model relating the level of the exposure (*X*) and the risk of the outcome (*Y*), which is binary (*Y* = 0,1). The causal risk ratio (CRR) is estimated using *G*, which is assumed to satisfy the conditions of an IV. The interpretation of the parameter as causal is subject to the IV assumptions; causal inferences are not obtained by statistical methodology, but rather by the use of untestable assumptions.

### Semiparametric methods: GMM and SMM

The GMM and SMM frameworks use estimating equations to obtain parameter estimates. Under the IV assumptions, the IV should be independently distributed from the value of the outcome if the exposure were set to 0, often written as the potential outcome *Y*(0). Independence between these random variables implies that the covariance between them is 0. The GMM/SMM estimate is taken as the value of the causal parameter, which equates the sample covariance between these variables in the data set to 0. We refer to the sample covariance as an “estimating function” rather than an objective function, because it is equated to 0 and not maximized or minimized. Parameter estimates can be obtained by evaluating the estimating function at various values of the parameter to find the value for which the estimating function is equal to 0 (a grid-search algorithm), or via an optimization routine.

Moment conditions for the estimation of the CRR using multiplicative structural models can be written in 2 equivalent ways owing to the 2 traditions (i.e., GMM and SMM) in which these approaches were developed. We consider 2 different sets of estimating equations depending on the error structure. First, assuming a structural model with multiplicative error, the GMM estimating equations are given by

(1) $$\sum\limits_i \,y_i \exp( - {\rm \beta}_1 x_i ) - {\rm \beta}_0 = 0$$

and

$$ \eqalign{\sum\limits_i \,g_i \,(y_i \exp( - {\rm \beta}_1 x_i ) - {\rm \beta}_0 ) = 0,} $$

where the parameter β_1_ is the log CRR, and the summation is across individuals indexed by *i* (19, 20). The parameter β_0_ corresponds to the probability of the outcome at *X* = 0, which should be similar in value to the prevalence of the outcome if *X* = 0 corresponds to a meaningful reference value for the exposure. We refer to this method as multiplicative generalized method of moments (MGMM).

Second, we can assume a structural model with additive error. The GMM estimating equations are

(2) $$\sum\limits_i \,y_i - \exp({\rm \beta }_{0^{\prime}} + {\rm \beta}_1 x_i ) = 0$$

and

$$\sum\limits_i \,g_i \,(y_i - \exp({\rm \beta }_{0^{\prime}} + {\rm \beta}_1 x_i )) = 0.$$

We refer to this method as the linear generalized method of moments (LGMM) (22, 23). If the models are correctly specified, and in the absence of confounding, both the MGMM and LGMM methods will consistently estimate the same CRR.

Alternatively, the MGMM estimator for β_1_ can be obtained in a SMM framework using a single estimating equation,

(3) $$\sum\limits_i \,y_i \exp( - {\rm \beta}_1 x_i )(g_i - \bar g) = 0$$

where $\bar g$ is the average value of *G* in the population. The sets of estimating equations 1 and 3 are equivalent when there are no covariates, in that the same estimate of β_1_ is obtained from both approaches (20). Hence, although we refer to the methods as MGMM and LGMM, either a GMM or an SMM estimating framework can be used to obtain the estimates.

### Parametric method: 2-stage method

Two-stage IV methods consist of 2 regression stages. In the first-stage regression, the exposure is regressed on the IV using linear regression. In the second-stage regression, the outcome is regressed on fitted values of the exposure taken from the first-stage regression. If the outcome is continuous, linear regression is usually used in the second-stage regression, and the 2-stage method is known as 2-stage least squares (24). If the outcome is binary, then log-linear regression can be used in the second stage to estimate a CRR. With a single IV, the 2-stage estimate is equal to the ratio (or Wald) estimate, calculated as the coefficient for the association of the IV with the outcome divided by the coefficient for the association of the IV with the exposure (8). In the ratio estimate, linear regression is used for the IV association with the exposure and linear or log-linear regression for the IV association with the outcome, as appropriate. The 2-stage method is a parametric estimation method, because a model for the outcome distribution is assumed for each individual.

Aside from in degenerate cases, log-linear and linear regression models (and other regression models based on an exponential family distribution) give unique parameter estimates, so there is no issue of lack of identification in the 2-stage method. The gradient of the log-likelihood function (i.e., the objective function) is a decreasing function of each of the parameters for all values of the data, and so it has a unique maximum. On the contrary, the estimating function in a GMM/SMM method is not guaranteed to be a monotonic (i.e., increasing or decreasing) function of the parameters, and so a unique parameter estimate is not always attained.

### Motivating example

**Figure 1.**
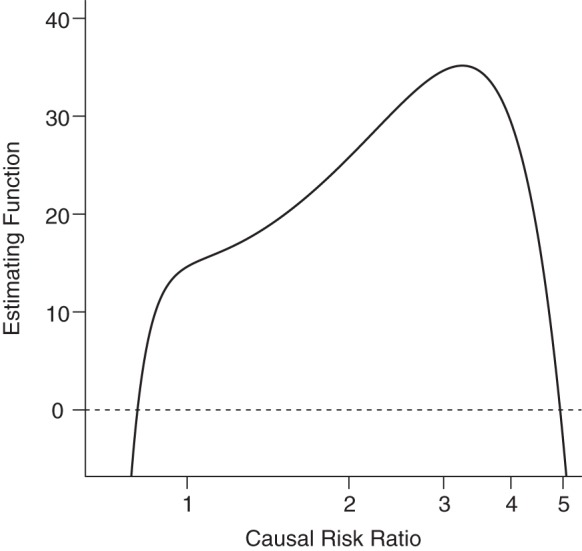
Estimating function for the example from Palmer et al. (20) demonstrating lack of identification. Two distinct parameter values for the causal risk ratio (0.81 and 4.95) satisfy the estimating equation $\sum\nolimits_i \,y_i \exp( - {\rm \beta}_1 x_i )(g_i - \bar g) = 0$, where $\bar g$ is the average value of *G* in the population.

In their paper, Palmer et al. (20) estimate the CRR for the effect of body mass index (BMI) (weight (kg)/height (m)^2^) on asthma risk using the MGMM method as exp(β_1_) = 0.81. However, as shown in Figure 1, the estimating function 3 for these data is equal to 0 at 2 separate values of the parameter exp(β_1_): 0.81 and 4.95 and is close to 0 between these values. In this case, the CRR is not uniquely identified by the moment conditions. This indicates that there is little information on the value of the CRR in the data and may result from the weakness of the IV.

A weak instrument is a variable satisfying the conditions of an IV, but which does not explain a substantial proportion of the variation in the exposure, usually measured by its F or R^2^ statistic in the regression of the exposure on the IV (25). Convention states that a weak instrument is one with an F statistic less than 10, although this definition was derived in the context of the 2-stage least squares method with a continuous outcome (10, 26). The use of this threshold for determining the weakness of an IV is often misleading and unhelpful (27), and is so for this case in particular, because the F statistic was 12.7. In fact, we are not aware of any investigation of the recommended strength for IVs for nonlinear outcome models. As well as lack of identification, weak instruments can lead to bias, instability in parameter estimates, and underestimated standard errors (28).

If the IV were truly independent of the exposure, then the estimating function 3 would be satisfied for all parameter values in the asymptotic limit (as the sample size tends toward infinity). This illustrates why a nonnull association between the IV and exposure is necessary for parameter identification.

### Simulation study

To examine the behavior of estimates from the GMM, SMM, and 2-stage methods with weak instruments, we simulate data from 5,000, 10,000, 20,000, and 50,000 individuals indexed by *i* from the following data-generating model:

(4) $$g_i \sim {{\cal N}}(0,{\rm \rho}^2 )$$

$$x_i \sim {{\cal N}}(g_i ,1 - {\rm \rho}^2 )$$

$$y_i \sim \hbox{Bernoulli}(\min{\{ }\exp({\rm \beta}_0 + {\rm \beta}_1 x_i ),1{\} }).$$

We generated 10,000 simulated data sets for each sample size. No confounding is incorporated into the data-generating model between *X* and *Y*, so as not to introduce additional complications due to differing assumptions in the methods relating to unobserved confounding variables. The instrumental variable is simulated as a continuous variable. In the context of Mendelian randomization, this can be interpreted as a weighted allele score. We set parameters β_0_ = −3 and β_1_ = 0.2, meaning that the prevalence of the outcome is 5%, and the CRR is exp(0.2), and we vary the strength of the instrument (ρ^2^ = 0.001, 0.002, 0.005, 0.01, 0.02, 0.03, 0.05, or 0.1). For example, when ρ^2^ = 0.02, the IV explains, on average, 2% of the variance in the exposure.

For the GMM and SMM methods, we investigate evidence for poor identification. This was assessed by evaluating the estimating function 3 at values of β_1_ from −50 to 50 at intervals of size between 0.1 and 1 and counting the number of times the sign of the expression changed. We performed a sensitivity analysis extending the resolution of parameter values considered; no substantial changes were observed in the findings. If the sign of the moment condition did not change, then no parameter value satisfied the moment condition. If the sign changed once, then the method gave a single parameter estimate (i.e., the parameter was identified). If the sign changed multiple times, then the method gave multiple parameter estimates, which satisfied the moment condition (i.e., the parameter was not identified).

### Applied example

We further illustrate our results with an applied example. We consider data from 1,805 female individuals from the Avon Longitudinal Study of Parents and Children (ALSPAC) on the causal effect of BMI, measured at age 7.5 years, on self-recalled early menarche, defined as before the age of 12 years. The study website contains details of all the data that are available through a fully searchable data dictionary (29). Ethical approval for the study was obtained from the ALSPAC ethics and law committee and the local research ethics committees. A positive causal effect of BMI on early menarche using IV methods has been previously observed (30). We use the following 3 IVs: 1) a variant (rs1558902) of the fat mass and obesity associated (*FTO)* gene, which is known to be associated with satiety (31); 2) the Speliotes score, which is an allele score (32) constructed using 32 variants shown to be associated with BMI in an independent data set at a genome-wide level of significance with weights derived from that data set (33); and 3) the Speliotes score with the *FTO* genetic variant excluded. The prevalence of early menarche was 30%, and the IVs explained 0.4%, 1.9%, and 1.4% of the variance in the exposure, respectively.

## RESULTS

### Simulation study

**Figure 2.**
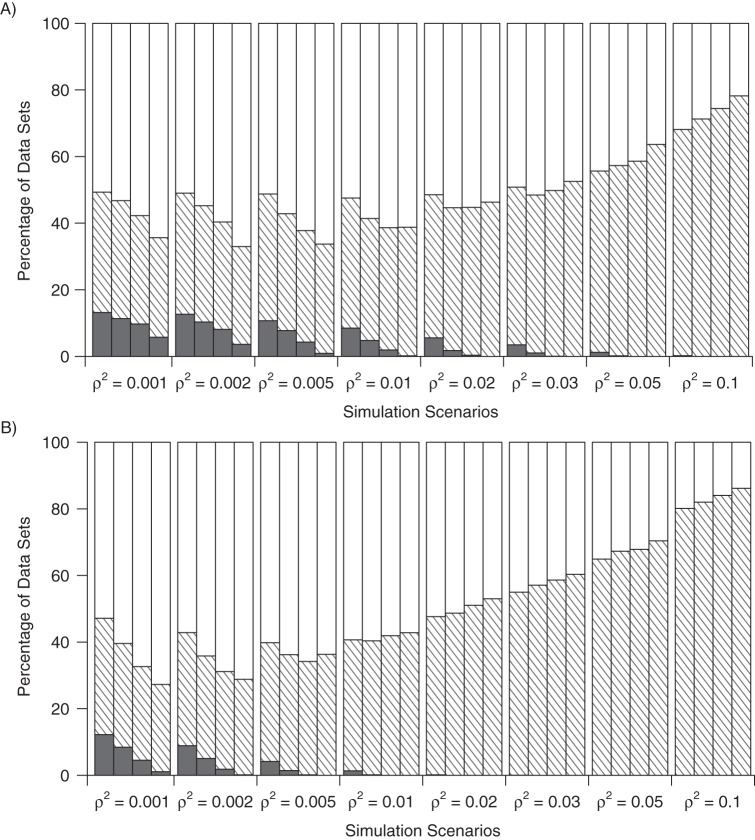
Percentage of simulated data sets with no solution (solid color), 1 solution (shaded), and multiple solutions (no color) from A) multiplicative generalized method of moments, and B) linear generalized method of moments methods with different strengths of instrument as measured by the squared correlation between the instrument and exposure (ρ^2^) and different sample sizes (*n*). For each value of ρ^2^, the first column is *n* = 5,000, the second column is *n* = 10,000, the third column is *n* = 20,000, and the fourth column is *n* = 50,000.

Results from the simulation study are displayed in Table 1 and Figure 2. For the GMM and SMM methods, we present the percentages of simulated data sets for which there was no solution, a unique solution, and multiple solutions to the estimating equations. The maximum number of solutions observed was 7. With 10,000 simulated data sets, the Monte Carlo standard error, representing the uncertainty in the results due to the limited number of simulations, was 0.5% or below.

**Table 1.** Percentage of Simulated Data Sets With No Solution, 1 Solution (Identified), and Multiple Solutions (Lack of Identification) From Multiplicative and Linear Generalized Method of Moments Methods With Different Strengths of Instrument as Measured by the Squared Correlation Between the Instrument and Exposure (ρ^2^) and the Mean F Statistic, and With Different Sample Sizes

ρ^2^	Mean F	MGMM Method			LGMM Method		
		No Solution, %	1 Solution, %	Multiple Solutions, %	No Solution, %	1 Solution, %	Multiple Solutions, %
*Scenario 1: 5,000 individuals*							
0.001	6.3	13.2	36.1	50.7	12.2	34.9	52.8
0.002	11.0	12.7	36.4	51.0	8.9	33.9	57.2
0.005	25.7	10.7	38.0	51.3	4.2	35.6	60.2
0.01	51.9	8.5	39.1	52.4	1.4	39.3	59.3
0.02	103.3	5.6	43.0	51.5	0.2	47.5	52.4
0.03	155.5	3.5	47.3	49.2	0.1	54.9	45.0
0.05	264.1	1.2	54.5	44.3	0.0	64.9	35.1
0.1	559.1	0.2	67.9	31.9	0.0	80.1	19.9
*Scenario 2: 10,000 individuals*							
0.001	10.8	11.3	35.4	53.2	8.4	31.2	60.4
0.002	20.9	10.3	34.9	54.8	5.1	30.8	64.2
0.005	51.1	7.7	35.1	57.2	1.4	34.8	63.8
0.01	100.4	4.8	36.6	58.6	0.2	40.2	59.6
0.02	205.3	1.8	42.8	55.4	0.0	48.7	51.3
0.03	310.1	1.1	47.4	51.6	0.0	57.1	42.9
0.05	528.3	0.2	57.1	42.7	0.0	67.3	32.7
0.1	1,110.4	0.0	71.3	28.7	0.0	82.0	18.0
*Scenario 3: 20,000 individuals*							
0.001	21.6	9.7	32.5	57.8	4.5	28.1	67.4
0.002	40.9	8.1	32.3	59.6	1.8	29.3	68.8
0.005	100.9	4.3	33.5	62.2	0.2	34.0	65.8
0.01	203.7	2.0	36.7	61.4	0.0	41.9	58.6
0.02	411.6	0.4	44.4	55.3	0.0	51.0	49.0
0.03	618.0	0.1	49.7	50.2	0.0	58.6	41.4
0.05	1,054.7	0.0	58.6	41.4	0.0	67.8	32.1
0.1	2,225.8	0.0	74.4	25.6	0.0	84.0	16.0
*Scenario 4: 50,000 individuals*							
0.001	49.7	5.8	29.9	64.3	1.1	26.2	72.7
0.002	100.6	3.6	29.3	67.0	0.2	28.7	71.2
0.005	251.9	0.9	32.8	66.3	0.0	36.3	63.7
0.01	506.8	0.2	38.6	61.2	0.0	42.8	57.2
0.02	1,019.1	0.0	46.3	53.7	0.0	53.0	47.0
0.03	1,544.5	0.0	52.5	47.5	0.0	60.4	39.6
0.05	2,631.3	0.0	63.6	36.4	0.0	70.4	29.6
0.1	5,565.3	0.0	78.2	21.8	0.0	86.2	13.8

Abbreviations: LGMM, linear generalized method of moments; MGMM, multiplicative generalized method of moments.

When a unique estimate was available, the MGMM and LGMM methods usually led to similar estimates, although estimates differed considerably in a sizeable minority of data sets. Mean estimates are not reported for the MGMM and LGMM methods because of a unique estimate not being available for a considerable proportion of simulated data sets. Estimates from the 2-stage method were highly variable, especially with the weakest of instruments, but they showed no clear pattern of bias (Web Table 1, Web Appendix 1 available at http://aje.oxfordjournals.org/). However, this is expected in the situation of no confounding.

The proportion of simulations having a unique GMM/SMM solution depended on the value of ρ^2^. For instruments with ρ^2^ ≤ 0.005, increasing the sample size did not lead to a greater proportion of simulations having a unique solution, because the proportion of simulations with multiple solutions also increased. For the weakest of IVs, the proportion even seems to decrease. This may represent data sets in which the correlation between the IV and exposure with a limited sample size is, by chance, greater than the expected value, for which the sample correlation decreases as the sample size increases. For instruments with ρ^2^ ≥ 0.02, the proportion of simulations with a unique solution increased as the sample size increased. The LGMM method gave no solution in fewer simulations than the MGMM method for all values of ρ^2^, and it gave a unique solution in more simulations with ρ^2^ ≥ 0.01. Poor identification was observed in spite of F statistics exceeding 1,000 in some cases.

### Large-sample behavior of GMM/SMM estimators

Additional simulations for a limited number of values of ρ^2^ (i.e., 0.005, 0.01, and 0.1) with 1 million participants are presented in Web Table 2 (Web Appendix 2). These provide evidence of poor identification at even larger sample sizes. Even if a unique solution from the GMM/SMM method is guaranteed in the asymptotic limit (i.e., as the sample size increases toward infinity), the probability of obtaining a unique solution increases so slowly that poor identification will be a relevant issue for all practically obtainable sample sizes when ρ ≤ 0.1, and potentially for stronger instruments.

In the linear case, the strength of an IV is usually measured by the F statistic. This means that any IV can be strengthened from a “weak” instrument to a “strong” instrument simply by increasing the sample size. On the contrary, for the log-linear case with a binary outcome using the GMM and SMM methods, the F statistic does not appear to be a relevant measure of instrument strength, and increases in the sample size do not seem to affect greatly the strength of the instrument, particularly when ρ^2^ ≤ 0.01.

### Absence of a solution to the estimating equations

A lack of a solution to the estimating equations with a single IV is an indication that there is not much information on the parameter of interest in the data set. In this case, a unique parameter estimate may still be obtained by minimizing an objective function based on the square of the estimating function 3. When there are multiple IVs, there is a separate estimating function for each IV. If there are more estimating functions than parameters, it is not possible, in general, for the estimating functions all to be equated to 0 simultaneously. Therefore, many automated software routines for GMM/SMM estimation do not solve the estimating equations, but instead minimize an objective function (24) (Web Appendix 3).

### Applied example

**Figure 3.**
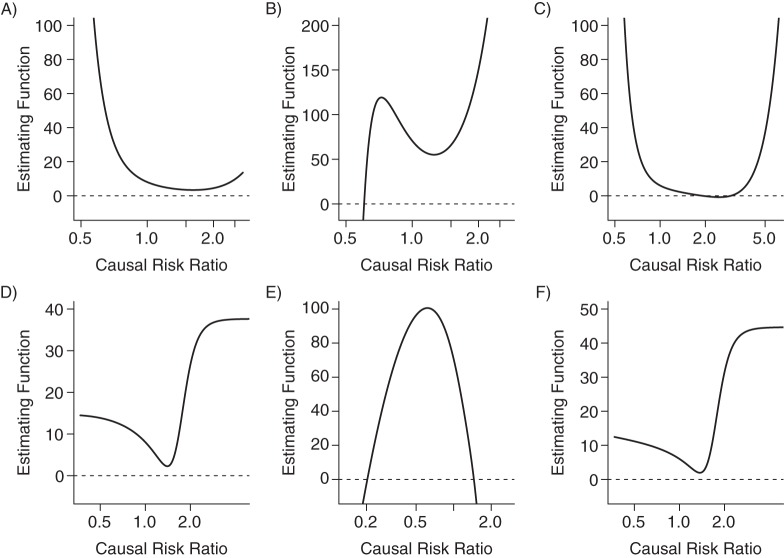
Estimating functions for the applied example from the multiplicative generalized method of moments method (in A, B, and C), and the linear generalized method of moments method (in D, E, and F) for the following 3 instruments: in A and D, a variant from the fat mass and obesity associated (*FTO*) gene; in B and E, the Speliotes score; and in C and F, the Speliotes score with the *FTO* genetic variant omitted. Avon Longitudinal Study of Parents and Children, 1991–1997.

Results from the applied example are given in Table 2. Supplementary details of the methods are provided in Web Appendix 4. The estimating functions for these data are shown in Figure 3. We see that the estimating function is not “well-behaved” in any of the cases: none of the functions is monotonic (either increasing for all values of the CRR or decreasing for all values). The only function with a single solution to the moment conditions is the MGMM estimating function using the *FTO* genetic variant, although this solution is far from the estimate reported by the automated optimization routine. The automated software command reports either 1 of the solutions to the estimating equations (MGMM method for Speliotes score without *FTO* genetic variant) or a minimum of the moment/objective function (all other cases).

**Table 2.** Estimates for the Effect of Body Mass Index^a^ on the Probability of Early Menarche From Multiplicative and Linear Generalized Method of Moments and 2-Stage Methods Using Different Instruments With Strength as Measured by the F Statistic, Avon Longitudinal Study of Parents and Children, 1991–1997

Instrument	F Statistic	MGMM Method		LGMM Method		2-Stage Method	
		Estimate	95% CI	Estimate	95% CI	Estimate	95% CI
*FTO* gene	7.8	1.28	NP^b^	1.45	1.00, 2.12	1.68	0.89, 3.17
Speliotes	34.1	1.64	NP^b^	1.40	1.35, 1.46	1.63	1.19, 2.21
Speliotes without *FTO* gene	25.4	1.91	0.42, 8.79	1.37	1.32, 1.42	1.61	1.12, 2.30

Abbreviations: CI, confidence interval; *FTO*, fat mass and obesity associated; LGMM, linear generalized method of moments; MGMM, multiplicative generalized method of moments; NP, not provided.

^a^ Weight (kg)/height (m)^2^.

^b^ The matrix used in calculating the standard errors of the parameter estimates was not invertible; no confidence interval was provided.

The MGMM method, in some cases, gave infinite confidence intervals, whereas the LGMM method using the Speliotes score (with and without the *FTO* genetic variant) gave a very narrow confidence interval. In contrast, the 2-stage method gave plausible results throughout, which were consistent across the different IVs. Additionally, we found that some of the MGMM and LGMM estimates were sensitive to the choice of starting values for the optimization routine and to whether the exposure was centered. When assessing the causal effect of the exposure on the outcome by testing for an association between the IV and the outcome, *P* values were similar to those for the 2-stage estimates (*P* = 0.06, 0.0003, and 0.002 for IV-outcome association, and *P* = 0.11, 0.002, and 0.009 for 2-stage estimate with each IV, respectively).

## DISCUSSION

In this paper, we have shown how parameter estimates in an instrumental variable analysis can suffer from this problem. Poor identification is most likely to occur in a nonlinear model, such as the binary outcome setting discussed, when the data do not contain much information on the parameter of interest. If the IV is weak (i.e., it explains a small proportion of the variance of the exposure in the data set), the causal effect may not be uniquely identified, and the estimating equations may not be satisfied for any parameter value, or they may be satisfied for multiple parameter values. Poor identification was observed in the semiparametric GMM and SMM frameworks, in which estimating equations are used for parameter estimation. Even when there is a unique parameter value, if the objective function is flat around the optimal value, then the data carry little information on the parameter of interest. This is known as weak identification and may lead to instability in parameter estimates, bias, and poor coverage properties of asymptotically derived confidence intervals.

The simulation study suggested that poor identification will be a common problem using GMM and SMM methods with binary outcomes, occurring in more than 50% of simulations with the MGMM method when the squared correlation between the IV and exposure (ρ^2^) was 0.02 or below for sample sizes up to 50,000 individuals and in a considerable proportion of simulations even when the squared correlation was 0.1 and the sample size was 1 million individuals. A similar model to equation 2 with a logistic link function was shown to result in poor convergence in a large number of simulated data sets by Vansteelandt et al. (34) and in a related model with a logistic link function in a small number (0.1%) of simulated data sets with a very strong instrument (ρ^2^ = 0.39) by Brumback et al. (35). For this reason, the assessment of identification should be a routine element of IV estimation using GMM and SMM methods.

Simulations suggest that the threshold for the strength of an IV, such that poor identification will be avoided, depends on the parameter ρ^2^, which is estimated in a given data set by the coefficient of determination *R*^2^ and does not greatly depend on the sample size for the range of values of ρ^2^ considered. This means that, for example, the use of GMM and SMM methods for Mendelian randomization when BMI is the exposure and the outcome is binary is likely to lead to problems of identification even when the sample size is very large, because *R*^2^ for the Speliotes score, comprising the 32 leading BMI-related variants, is approximately 1.4% in the larger data set used to derive the Speliotes score (33). The F statistic is not a relevant measure of instrument strength in this context.

Lack of identification indicates that the data do not carry much information on the causal effect of interest. If investigators discover lack of identification in an applied context, they should consider not providing a point estimate for the causal effect and simply reporting the test for association between the IV and the outcome to establish a causal effect of the exposure on the outcome (which should be reported in any case) (36). If they consider using an alternative estimation technique, such as a fully parametric method, it should be made clear that this makes use of stronger untestable assumptions in order to obtain identification. An alternative approach is to augment the set of IVs by transforming the IVs to obtain further estimating equations, which may lead to a unique minimum of the GMM/SMM objective function. However, the use of large numbers of IVs may lead to weak instrument bias, particularly when the IVs are only weakly associated with the exposure, and it should not be performed indiscriminately or on a post hoc basis.

In conclusion, estimates from semiparametric GMM and SMM methods for instrumental variable analysis may suffer from a lack of identification, meaning that parameter estimates are not unique. Consequently, estimates from automated software routines for GMM or SMM estimation can be misleading. We therefore recommend that applied investigators wanting to use a GMM or SMM approach should plot the relevant estimating function for a large range of values of the parameter of interest to check if there is a unique solution to the estimating equations.

## Supplementary Material

Web Material
