# Supplementary material for: Lack of Identification in Semiparametric Instrumental Variable Models With Binary Outcomes
Source: Am J Epidemiol. 2014 May 23;180(1):111–9. doi: 10.1093/aje/kwu107 (PMC4070936; doi:10.1093/aje/kwu107)
Supplement: Web Material [file supp_180_1_111__index.html]

Lack of Identification in Semiparametric Instrumental Variable Models With Binary Outcomes — Web Material 

# Lack of Identification in Semiparametric Instrumental Variable Models With Binary Outcomes

## Web Material

Web Material

**Files in this Data Supplement:**

- Web Material - Pdf file
