# Supplementary material for: Lack of Identification in Semiparametric Instrumental Variable Models With Binary Outcomes
Source: Am J Epidemiol. 2014 May 23;180(1):111–9. doi: 10.1093/aje/kwu107 (PMC4070936; doi:10.1093/aje/kwu107)
Supplement: Web Material [file supp_kwu107_kwu107supp.pdf]

Web Appendix 1

Estimates from two-stage method

Estimates using the two-stage IV method were calculated in the simulation study from the main paper. Mean, median and standard deviations of estimates across simulations are given in Web Table 1. Estimates with the weakest of IVs are difficult to interpret due to the large Monte Carlo (simulation) variance. Indeed, the expected value of the two-stage estimator with a single instrumental variable is undefined for all finite sample sizes, due to the small but finite probability that the estimated association between the instrument variable and the exposure is close to zero, and so the IV estimate is arbitrarily large [1]. Even summarized results with the weakest of IVs were highly variable, and the mean estimate across simulations was sometimes large and positive, sometimes negative, and sometimes close to the true value of $\beta_1 = 0.2$. With stronger IVs, mean and median estimates across simulations were close to the true parameter value.

As there was no confounding in the data-generating model, systematic weak instrument bias is not expected. However, this limited simulation study should not be interpreted as providing evidence for the behavior of the two-stage method with weak instruments. A more detailed investigation with a more realistic data-generating model incorporating confounding would be needed to make such claims. Such an investigation is beyond the scope of this paper. Bias of the two-stage method with a continuous outcome has been discussed previously [1, 2, 3], as has the bias with a binary outcome and a logistic model of association [3, 4, 5]; similar results are expected

with a binary outcome and a log-linear model of association.

ρ^2	Mean F	Mean	Median	SD	Mean F	Mean	Median	SD
	5000 individuals				10 000 individuals			
0.001	6.3	0.504	0.214	28.325	10.8	-0.187	0.192	39.380
0.002	11.0	0.222	0.188	2.110	20.9	0.188	0.184	1.058
0.005	25.7	0.202	0.207	0.944	51.1	0.205	0.200	0.631
0.01	51.9	0.203	0.205	0.633	100.4	0.193	0.191	0.444
0.02	103.3	0.197	0.201	0.439	205.3	0.192	0.190	0.305
0.03	155.5	0.201	0.203	0.359	310.1	0.201	0.203	0.248
0.05	264.1	0.194	0.195	0.275	528.3	0.200	0.203	0.193
0.1	559.1	0.199	0.200	0.194	1110.4	0.200	0.199	0.136
	20 000 individuals				50 000 individuals			
0.001	21.6	0.215	0.208	1.063	49.7	0.208	0.198	0.635
0.002	40.9	0.188	0.181	0.711	100.6	0.198	0.198	0.439
0.005	100.9	0.200	0.200	0.438	251.9	0.200	0.201	0.278
0.01	203.7	0.202	0.205	0.308	506.8	0.200	0.202	0.195
0.02	411.6	0.202	0.202	0.218	1019.1	0.202	0.202	0.137
0.03	618.0	0.203	0.202	0.178	1544.5	0.199	0.200	0.112
0.05	1054.7	0.198	0.199	0.136	2631.3	0.201	0.201	0.087
0.1	2225.8	0.200	0.198	0.097	5565.3	0.201	0.201	0.061

Web Table 1: Mean and median estimates of $\beta_1 = 0.2$ and standard deviation (SD) of estimates across simulations from two-stage method with different strengths of instrument as measured by the squared correlation between the instrument and exposure (ρ^2) and the mean F statistic, and with different sample sizes.

Web Appendix 2

Increasing the sample size

Simulations in the main body of the paper suggest that the behavior of the probability of obtaining a unique solution from the MGMM and LGMM methods as the sample size increases depends on the value of the squared correlation between the IV and the exposure (ρ^2). In particular, when the IV is not strong, the probability does not seem to increase as the sample size increases. To investigate whether this pattern continues for very large sample sizes, we simulate data on 1 000 000 individuals according to the same data-generating model (4) and parameters as in the simulation study. Three values of $\rho^2 = 0.005, 0.01, 0.1$ are considered. For computational reasons, only 1000 datasets were analyzed.

Results are displayed in Web Table 2. This provides another datapoint supporting the hypothesis that the large sample behavior of the MGMM and LGMM estimators depends mostly on the value of ρ^2. The probability of obtaining a unique solution using the MGMM method with a sample size of 1 000 000 is less than that for a sample size of 5 000 when $\rho^2 = 0.005$.

		MGMM method			LGMM method		
ρ^2	Mean F	No solution	One solution	Multiple solutions	No solution	One solution	Multiple solutions
Scenario 1: 5000 individuals							
0.005	25.7	10.7%	38.0%	51.3%	4.2%	35.6%	60.2%
0.01	51.9	8.5%	39.1%	52.4%	1.4%	39.3%	59.3%
0.1	559.1	0.2%	67.9%	31.9%	0.0%	80.1%	19.9%
Scenario 4: 50 000 individuals							
0.005	251.9	0.9%	32.8%	66.3%	0.0%	36.3%	63.7%
0.01	506.8	0.2%	38.6%	61.2%	0.0%	42.8%	57.2%
0.1	5565.3	0.0%	78.2%	21.8%	0.0%	86.2%	13.8%
Scenario 5: 1 000 000 individuals							
0.005	5024	0.0%	34.2%	65.8%	0.0%	38.2%	61.8%
0.01	10099	0.0%	41.9%	58.1%	0.0%	47.8%	52.2%
0.1	111099	0.0%	85.7%	14.3%	0.0%	91.5%	8.5%

Web Table 2: Percentage of simulated datasets with no solution, one solution (identified), and multiple solutions (lack of identification) from multiplicative and linear generalized method of moments methods with different strengths of instrument as measured by the squared correlation between the instrument and exposure (ρ^2) and the mean F statistic, and with large sample size of 1 000 000 individuals.

Abbreviations: LGMM, linear generalized method of moments; MGMM, multiplicative generalized method of moments.

Web Appendix 3

Absence of a solution to the estimating equations

If the estimating functions are expressed as a vector $\mathbf{g}(\boldsymbol{\beta})$, then the usual objective function is:

$$J(\boldsymbol{\beta}) = \mathbf{g}(\boldsymbol{\beta})^T W \mathbf{g}(\boldsymbol{\beta}) \tag{A.1}$$

where W is a weighting matrix, assumed to be of full rank. This is a quadratic form, and so has a unique minimum. The choice of W affects the efficiency of estimates, but not the consistency [6]. The objective function will be equal to zero if and only if the estimating functions are all zero, hence the simulation study to investigate identification performed in this paper is agnostic to the choice of weighting matrix. In any case, with a single IV, the choice of weighting matrix is moot, as the first moment condition in the MGMM or LGMM methods can always be equated to zero by altering the intercept term β_0. Efficient estimates for MGMM with multiple IVs have been discussed previously [7], but are beyond the scope of this paper.

The minimizer of the objective function may be a valid estimate (that is, consistent for the target parameter of interest) even when it does not solve the estimating equations, if the values of the estimating equations at the estimate tend to zero as the sample size tends to infinity [8]. However, there is no guarantee that the solution will be unique.

Web Appendix 4

Supplementary methods for applied example

Here we provide additional details of how the applied analyses in the main paper was undertaken. GMM/SMM analyses were performed using the *gmm* command Stata 12 [9]; this command minimizes an objective function similar to equation (A.1). The weighting matrix used is derived from a two-step estimation procedure, in which the first step uses the identity weighting matrix to obtain a parameter estimate which is used to construct the second-step weighting matrix [10]. Starting values of $(0, 0)$ were taken and the exposure was centered throughout (this means that the reference value of the exposure where $X = 0$ was 16.4 kg/m^2). A two-stage method was undertaken, using linear regression in the first stage and log-linear (Poisson) regression in the second stage [11]. In the two-stage method, robust standard errors to account for uncertainty in the first-stage regression were not used, as their use decreased standard errors, rather than increasing them. We also assessed the causal effect of the exposure on the outcome by testing for an association between the IV and the outcome using log-linear regression. The assessment of a causal effect requires the weakest assumptions of any of the methods as the test requires no assumption on the distribution of the exposure. The stronger distributional assumptions of the two-stage method were satisfied in the simulation study, but cannot be fully tested in the applied example. We examined the distribution of the exposure at various values of the IV; it was close to normal.

References

[1] Burgess S, Thompson S. Bias in causal estimates from Mendelian randomization studies with weak instruments. *Statistics in Medicine* 2011;30(11):1312–1323.

[2] Burgess S, Thompson S, CRP CHD Genetics Collaboration. Avoiding bias from weak instruments in Mendelian randomization studies. *International Journal of Epidemiology* 2011;40(3):755–764.

[3] Burgess S, Thompson S. Improvement of bias and coverage in instrumental variable analysis with weak instruments for continuous and binary outcomes. *Statistics in Medicine* 2012;31(15):1582–1600.

[4] Harbord R, Didelez V, Palmer T, Meng S, Sterne J, Sheehan N. Severity of bias of a simple estimator of the causal odds ratio in Mendelian randomization studies. *Statistics in Medicine* 2013;32(7):1246–1258.

[5] Burgess S, CHD CRP Genetics Collaboration. Identifying the odds ratio estimated by a two-stage instrumental variable analysis with a logistic regression model. *Statistics in Medicine* 2013;32(27):4726–4747.

[6] Vansteelandt S, Goetghebeur E. Causal inference with generalized structural mean models. *Journal of the Royal Statistical Society: Series B (Statistical Methodology)* 2003;65(4):817–835.

[7] Bowden J, Vansteelandt S. Mendelian randomisation analysis of case-control data using structural mean models. *Statistics in Medicine* 2011;30(6):678–694.

[8] Wooldridge J. *Introductory econometrics: A modern approach.* South-Western, Nashville, TN, 2009.

[9] StataCorp. *Stata Statistical Software: Release 12.* College Station, TX, 2011.

[10] White H. A heteroskedasticity-consistent covariance matrix estimator and a direct test for heteroskedasticity. *Econometrica: Journal of the Econometric Society* 1980;48(4):817–838.

[11] Palmer T, Sterne J, Harbord R, Lawlor D, Sheehan N, Meng S, et al. Instrumental variable estimation of causal risk ratios and causal odds ratios in Mendelian randomization analyses. *American Journal of Epidemiology* 2011;173(12):1392–1403.
